# Type I interferons modulate autophagy to shape gemcitabine response in pancreatic cancer cells

**DOI:** 10.1002/2211-5463.70342

**Published:** 2026-09-15

**Authors:** Lucy E. Bonilla, Martín M. Ledesma, Santiago A. Behr, Matías A. Pibuel, María L. Palanek, Daniel H. Grasso, Maria N. Garcia, Juan Garona, Silvina L. Lompardía, Elida Álvarez, Daniela L. Papademetrio

**Affiliations:** ^1^ Cátedra de Inmunología, Departamento de Microbiología, Inmunología, Biotecnología y Genética, Facultad de Farmacia y Bioquímica Universidad de Buenos Aires Argentina; ^2^ Instituto de Estudios de la Inmunidad Humoral (IDEHU), UBA – CONICET Buenos Aires Argentina; ^3^ Unidad de Conocimiento Traslacional, Hospital de Alta Complejidad del Bicentenario Esteban Echeverría Buenos Aires Argentina; ^4^ Centro de Investigaciones en Biomedicina traslacional CIBiMeT, CONICET ‐ Hospital de Alta Complejidad del Bicentenario Esteban Echeverría Buenos Aires Argentina; ^5^ Cátedra de Fisiopatología, Departamento de Ciencias Biológicas, Facultad de Farmacia y Bioquímica Universidad de Buenos Aires Argentina; ^6^ Centro de Medicina Traslacional (CEMET) Unidad de Investigaciones Biomédicas en Cáncer (IBioCAN, Unidad N°6), Hospital de Alta Complejidad en Red El Cruce “Dr. Néstor Carlos Kirchner” S.A.M.I.C. Florencio Varela, Buenos Aires, Argentina.; ^7^ Centro de Oncología Molecular y Traslacional (COMTra) Unidad de Oncología Traslacional, Universidad Nacional de Quilmes. Quilmes, Buenos Aires, Argentina.

**Keywords:** autophagy, chemoresistance, gemcitabine, IFNα2b, IFNβ1a, pancreatic cancer

## Abstract

Pancreatic ductal adenocarcinoma (PDAC) is an aggressive malignancy characterized by poor prognosis and limited response to gemcitabine, the standard first‐line chemotherapy. One major contributor to chemoresistance is autophagy, a process frequently upregulated in PDAC. In this study, we examined the ability of type I interferons (IFNα2b and IFNβ1a) to modulate autophagy and disturb tumor cell resistance to gemcitabine. PDAC cells were treated with increasing concentrations of IFNα2b or IFNβ1a, and cell proliferation was assessed by [^3^H]‐thymidine incorporation. Apoptosis was evaluated by TUNEL staining following treatments with interferons and/or gemcitabine. STAT1 phosphorylation was analyzed as a marker of downstream type I interferon signaling. Autophagy was analyzed by western blot for LC3B, Beclin‐1, Bcl‐XL and p62/SQSTM1, and by quantifying autophagic flux using mCherry‐EGFP‐LC3B‐transfected cells in the presence or absence of lysosomal inhibitors. We found that IFNα2b promoted autophagic flux and reduced gemcitabine‐induced cell death, indicating a cytoprotective role. In contrast, IFNβ1a reduced autophagosome formation and significantly enhanced cell death, without clear evidence of altering autophagic flux. Both interferons induced STAT1 phosphorylation, confirming engagement of downstream type I interferon signaling. Our findings highlight the contrasting roles of IFNα2b and IFNβ1a in the regulation of autophagy and gemcitabine response and suggest that IFNβ1a, by reducing autophagosome formation, may sensitize PDAC cells to chemotherapy. These findings identify IFNβ1a as a potential chemosensitizing agent in PDAC and provide a rationale for further evaluating combinations of IFNβ1a, gemcitabine, and autophagy‐targeting strategies to overcome chemoresistance.

## Graphical abstract

Type I interferons differentially modulate autophagy and the response of pancreatic cancer cells to gemcitabine. IFNα2b stimulates autophagic flux and protects cells from gemcitabine‐induced cell death, contributing to chemoresistance. In contrast, IFNβ1a inhibits autophagosome formation and enhances gemcitabine‐induced cell death, resulting in chemosensitization.

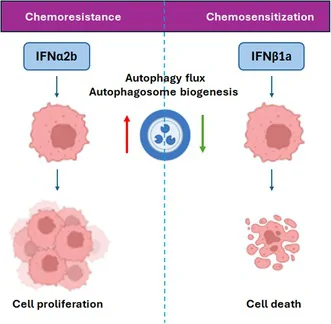

**Abbreviations**
[^3^H]TdR: tritiated thymidine
3‐MA: 3‐methyladenine
AKT: Protein Kinase B
ATCC: American Type Culture Collection
CQ: chloroquine
DAPI: 4′,6‐diamidino‐2‐phenylindole dihydrochloride
DMEM: Dulbecco's modified Eagle medium
EC50: half maximal effective concentration
EGFP: enhanced green fluorescent protein
FBS: fetal bovine serum
HCC: hepatocellular carcinoma
HEPES: 4‐(2‐hydroxyethyl)‐1‐piperazineethanesulfonic acid
IC25 / IC50: inhibitory concentration 25% / 50%
IFNAR‐1 / IFNAR‐2: interferon alpha/beta receptor subunit 1 / 2
IFN‐I: type I interferons
IFNα2b: interferon alpha 2b
IFNβ1a: interferon beta 1a
JAK/STAT: Janus kinase / Signal Transducer and Activator of Transcription
LC25: lethal concentration 25%
LC3B / LC3‐II: microtubule‐associated protein 1A/1B‐light chain 3 isoform B / lipidated form (II)
MAPK: mitogen‐activated protein kinase
MHC: major histocompatibility complex
mTORC1 / mTORC2: mechanistic target of rapamycin complex 1 / 2
PBS: phosphate‐buffered saline
PDAC: pancreatic ductal adenocarcinoma
PI3K: phosphatidylinositol 3‐kinase
PVDF: polyvinylidene difluoride
R: R statistical software
RFP: red fluorescent protein
SD: standard deviation
TBS: Tris‐buffered saline
TUNEL: terminal deoxynucleotidyl transferase dUTP nick end labeling
VCR: vincristine

Pancreatic ductal adenocarcinoma (PDAC) is one of the leading causes of cancer‐related death and remains a highly lethal malignancy despite advances in surgical and systemic therapies [1]. The prognosis of patients with pancreatic cancer is particularly poor even after curative resection. Tumor recurrence occurs in more than half of patients, and the estimated 5‐year survival rate is not greater than 20% [2, 3]. Gemcitabine (2′,2′‐difluorodeoxycytidine), a cell cycle–specific chemotherapeutic agent that interferes with DNA synthesis and ribonucleotide reductase activity, remains the standard treatment for PDAC; however, fewer than 20% of patients respond, underscoring the limited efficacy of current therapeutic approaches [4, 5].

A key biological feature of PDAC that contributes to its aggressive behavior, and therapeutic resistance is the high level of basal autophagy observed in pancreatic tumors and cancer cell lines. Autophagy is a highly conserved lysosomal degradation process responsible for the turnover of intracellular components, including damaged organelles and misfolded proteins [6]. Among the three types of autophagy—microautophagy, chaperone‐mediated autophagy, and macroautophagy—the latter (hereafter referred to as autophagy) plays a central role in cancer biology [7]. The effects of autophagy on pancreatic carcinogenesis and progression differ depending on the stage and context. In the early stage, autophagy hinders the development of preneoplastic lesions, whereas in the progression stage, autophagy promotes tumor growth. This dual role of autophagy makes it a complex therapeutic target [8, 9, 10]. Consistent with this notion, pancreatic tumors and PDAC cell lines exhibit elevated basal autophagy, as evidenced by increased LC3B expression (a membrane‐associated marker for all stages of autophagy) and a higher number of autophagosomes per cell, supporting the concept that autophagy functions as a survival mechanism in established pancreatic tumors [4, 7, 11]. Because autophagy supports tumor cell survival in advanced PDAC, its modulation has emerged as a relevant therapeutic strategy. A growing body of evidence indicates that chemotherapeutic agents and radiation can further induce a cytoprotective form of autophagy in tumor cells, thereby limiting treatment efficacy. This protective effect becomes apparent when autophagy is pharmacologically inhibited—using agents such as chloroquine, bafilomycin, 3‐methyladenine, or ammonium chloride—or genetically suppressed through the silencing of autophagy‐related genes, including Beclin‐1 and ATG5, ATG7, or ATG12. Under these conditions, tumor cells become more sensitive to therapy, typically through the reactivation of apoptotic cell death [4, 12, 13]. In line with these observations, we previously demonstrated that autophagy inhibition is critical for improving the response of MIAPaCa‐2 and PANC‐1 cells to gemcitabine, both *in vitro* and *in vivo* [4]. Together, these findings indicate that targeting autophagy may enhance therapeutic efficacy in PDAC, particularly in contexts in which therapy‐induced autophagy exerts a cytoprotective role [14].

The regulation of autophagy is closely integrated with intracellular signaling networks that govern cell growth and survival. Among these, mTORC1 functions as a central regulator of autophagy by linking nutrient availability to proliferative signals. mTORC1 activity is controlled by several upstream pathways, including the PI3K–AKT axis, which can be modulated by type I and type II interferons [15]. Interferons (IFN) have been shown to induce AKT activation in different cell types [16, 17], and AKT signaling has been proposed to attenuate IFN‐induced responses by interfering with apoptosis and promoting cell survival [18, 19]. Despite these observations, the functional relevance of AKT–mTOR signaling in the context of IFN responses, particularly with respect to autophagy regulation in PDAC, remains poorly understood.

Type I interferons (IFN‐I) are a family of cytokines originally characterized for their antiviral activity but increasingly recognized for their immunomodulatory and antitumoral functions [20, 21]. This family includes IFN‐α, IFN‐β, IFN‐ε, IFN‐κ, IFN‐ω, and IFN‐τ, with IFN‐α and IFN‐β being the most extensively studied members [22]. All IFN‐I signal through the type I interferon receptor complex (IFNAR1/IFNAR2), leading to activation of the canonical JAK/STAT pathway [23, 24, 25] as well as noncanonical signaling cascades, including p38 MAPK, PI3K–AKT, and mTORC1/mTORC2 pathways [15, 26, 27, 28]. It has been proven that IFNα2b has antitumor properties when combined with other chemotherapeutic agents for treating PDAC and other malignant neoplasms and has been associated with a substantial increase in 5‐year survival in the adjuvant setting [29, 30, 31]. In contrast, although IFNβ has shown growth‐inhibitory effects *in vitro* when combined with gemcitabine, its clinical relevance in pancreatic cancer remains undefined, and this combination appears to be predominantly cytostatic rather than cytotoxic [32].

Notably, activation of the canonical IFN signaling pathway, evidenced by increased phosphorylation of STAT1, STAT2, and STAT3, has been demonstrated in MIAPaCa‐2 and PANC‐1 cells [32]. However, whether type I interferons differentially regulate autophagy in PDAC cells, and how such regulation influences the cellular response to gemcitabine, has not yet been elucidated. In this context, the present study aimed to evaluate the effects of IFNα2b and IFNβ1a on the autophagy process and to assess how these effects influence the susceptibility of PDAC cells to gemcitabine‐induced cytotoxicity.

## Materials and methods

### Materials

IFNα2b and IFNβ1a were obtained from BioSidus (Buenos Aires, Argentina). Acridine orange, 4′,6‐Diamidino‐2‐phenylindole dihydrochloride (DAPI) and 3‐MA were purchased from Sigma‐Aldrich (St. Louis, MO, USA). Gemcitabine was kindly provided by Richmond (Buenos Aires, Argentina). Vincristine (VCR) was obtained from Filaxis (Buenos Aires, Argentina), and DMEM, penicillin, and streptomycin were purchased from Invitrogen (Invitrogen Argentina S.A., Buenos Aires, Argentina). Fetal bovine serum (FBS) was purchased from Internegocios S. A. (Buenos Aires, Argentina). Plasmid pRFP‐LC3 was generously supplied by Dr. Noboru Mizushima (Department of Physiology and Cell Biology, Tokyo Medical and Dental University, Tokyo, Japan) and Dr. Tamotsu Yoshimori (Department of Cellular Regulation, Research Institute for Microbial Diseases, Osaka University, Suita, Osaka, Japan). pBABE‐puro mCherry‐EGFP‐LC3B was a gift from Jayanta Debnath (Addgene plasmid #22418; http://n2t.net/addgene:22418; RRID:Addgene22418).

### Cell culture and viability

MIAPaCa‐2 (RRID:CVCL_0428), clone CRL‐1420 and PANC‐1 (RRID:CVCL_0480), and clone CRL‐1469 cells were obtained from the American Type Culture Collection (ATCC). The cells were cultured in DMEM containing 10% heat‐inactivated FBS, 2 mm L‐glutamine, 20 mm HEPES buffer, 100 IU/mL penicillin, and 150 μg/mL streptomycin at 37 °C in a humidified incubator with 5% CO_2_ and tested for Mycoplasma every 3 months via PCR (Abcam cat# ab289834). Cells at fewer than 20 passages were used for the experiments described. Cell viability was determined by trypan blue exclusion.

### Assessment of cell proliferation

The sensitivity of the cell line to increasing doses of either IFNα2b or IFNβ1a (10–10 000 IU/mL) was determined by culturing 5 × 10^4^ cells/ml at 37 °C in a 5% CO_2_ atmosphere for 24, 48, and 72 h. The cells were pulsed with 1 μCi [^3^H]TdR (DuPont/NEN Products, Boston, MA, USA) for 18 h. Cultures were performed in 96‐well flat microtiter plates. After incubation, the cells were harvested via a semiautomatic method. The incorporated [^3^H]TdR was measured in a liquid scintillation beta counter (Beckman/PerkinElmer, Waltham, MA, USA). The results were calculated from the mean counts per minute (cpm) of [^3^H]TdR incorporated in triplicate cultures. The percentage of cell proliferation was calculated as follows:

$$\%\text{Cell proliferation}=\frac{\mathrm{cpm}\;\text{treated cells}}{\mathrm{cpm}\;\text{basal control}}\times 100$$

The untreated cells used as the basal control represented 100% proliferation. The cell viability at the beginning of the experiment was greater than 90%, as assessed by Trypan blue exclusion. Each experiment was carried out at least three times, and similar results were obtained.

### Apoptotic assessment

Terminal deoxynucleotidyl transferase‐mediated dUTP nick end labeling (TUNEL) was carried out. Briefly, the cells were incubated alone in DMEM containing 10% FBS with IFN‐I and/or gemcitabine for 72 h. The cells were resuspended and washed once with ice‐cold phosphate‐buffered saline (PBS) and fixed in 4% buffered paraformaldehyde. The TUNEL assay was carried out via the DeadEnd Fluorometric TUNEL System Kit (Promega Corporation, Madison, WI, USA) following the manufacturer's recommendations. Images from triplicate samples were automatically obtained with an EVOS M7000 Microscope (Thermo Fisher Scientific) at 200× magnification. Images were processed with FIJI (imagej2) software. A minimum of 2000 cells were counted for each condition. Cells with pyknotic nuclei and dark green fluorescence staining were scored as positive. The cells were stained with DAPI as a counterstain. The percentage of TUNEL‐positive cells was calculated as follows:

$$\%\text{positive cells}=\frac{\text{number of green fluorescent cells}}{\text{number of DAPI stained cells}}\times 100$$

### Total protein extracts

The cells (1 × 10^7^) were lysed with hypotonic buffer (20 mm Tris pH 8.0, 150 mm NaCl, 100 mm NaF, 10% glycerol, 2% Nonidet P‐40) and the protease inhibitor cocktail P8340 from Sigma‐Aldrich for 30 min at 4 °C, followed by centrifugation at 13 000× ***g*** for 30 min. The extracts were then stored at −86 °C until use. The protein concentration was determined via the Bradford method.

### Western blot

Equal amounts of protein were loaded into each sample, separated by SDS/PAGE, and transferred onto PVDF membranes (GE Healthcare, Little Chalfont, UK). The membranes were blocked with 3% nonfat dry milk in TBS or 0,1% tween 20 in TBS‐BSA 3%, overnight at 4 °C. The membranes were then incubated with antibodies against STAT1 phosphorylated (Tyr701) (Cat # 9167), STAT1 (Cat # 14995) andLC3‐B (Cat #2775) from Cell Signaling Technology, Danvers, MA, USA, and Beclin‐1 (sc‐48 341), Bcl‐XL (sc‐7195), p62/SQSTM1 (sc‐48 402) and β‐actin (H‐196, cat sc‐7210) from Santa Cruz Biotechnology, Inc., Dallas, TX, USA, overnight at 4 °C. Horseradish peroxidase‐labeled anti‐rabbit (sc‐2030) and anti‐goat (sc‐2033) secondary antibodies were obtained from Santa Cruz (Dallas, Texas, USA), added at a ratio of 1 : 8000, and incubated for 1.5 h at 37 °C. Immunoblots were analyzed via a chemiluminescent detection system (western blotting luminol reagent, Santa Cruz Biotechnology, Inc.). Autoradiography images were obtained with a digital camera (Olympus D‐510 Zoom; Olympus Corporation, Tokyo, Japan) and subjected to densitometry analysis with FIJI software.

### Acridine orange staining

MIAPaCa‐2 and PANC‐1 cells were cultured in DMEM containing 10% FBS and treated with 1000 IU/mL IFNα2b or IFNβ1a for 24 h. Cells were then stained with acridine orange 1 μg/mL for 10 min and examined by fluorescence microscopy. Acidic vesicular organelles were evaluated qualitatively based on the fluorescence pattern observed under each experimental condition.

### RFP‐LC3 transfection and fluorescence microscopy

MIAPaCa‐2 and PANC‐1 cells were transfected with the RFP‐LC3 plasmid using the TransIntro PL (TransGen Biotech) transfection reagent, according to the manufacturer's instructions. After 24 h, cells were treated with 1000 IU/mL IFNα2b or IFNβ1a for 24 h. Fluorescence images were acquired using a EVOS M700 imaging system. RFP‐LC3‐positive puncta were evaluated qualitatively under each treatment condition. Nuclei were counterstained with DAPI.

### Autophagy flux assay

Autophagy flux was evaluated by western blot and image cytometry by transfecting cells with the plasmid pBABE‐puro‐mCherry‐EGFP‐LC3B (Addgene #22418), which expresses chimeric LC3 with GFP (green) and mCherry (red). For the first strategy, cells were cultured with 1000 IU/mL IFNα2b or IFNβ1a for 24 h and/or 10 μm VCR, a microtubule network inhibitor that blocks transport of autophagosomes to lysosomes, for the last 6 h. Protein extracts were obtained as detailed in the corresponding section. For the second strategy, 75% confluent MiaPaCa‐2 and PANC‐1 cells were transfected with pBABE‐puro‐mCherry‐EGFP‐LC3B (Addgene plasmid #22418) via the TransIntro PL (TransGen Biotech) according to the manufacturer's instructions. The transfected cells were incubated for 24 h in DMEM supplemented with 10% FBS in 24‐well plates and then treated with 1000 UI/ml IFNα2b or IFNβ1a and/or 25 μm CQ, a lysosomal pH‐neutralizing agent. Photographs were taken with an EVOS M700 imaging system and analyzed with FIJI software (imagej2).

### Statistical analysis

To analyze the dose–response curves, we performed nonlinear regression via the log–logistic function in the drc R package [33]. A four‐parameter model estimates two parameters, the EC50 and the Hill coefficient, while the maximum value (max_value) and minimum value (min_value) are set to 100% and the minimum experimental value, respectively. Finally, we use these estimated and set parameters to calculate the fitted survival or viability values for each concentration (Cc) value with the following equation:

$$\begin{array}{l} \text{Viability}|\text{Cell proliferation}\\ \;=\frac{\min\_\text{value}+\left(\max\_\text{value}-\min\_\text{value}\right)}{1+{\left(\mathrm{Cc}/\mathrm{EC}50\right)}^{\text{Hill}}} \end{array}$$

IC25 and LC25 refer to the ‘quarter‐maximal inhibitory or lethal concentrations’ for each drug against the biological processes or functions tested (such as cell proliferation and cell death) and were calculated via the following equation:

$$\;\mathrm{IC}25/\mathrm{LC}25=\begin{array}{l} \exp\left(\log\left(\mathrm{EC}50\right)+(\frac{1}{\text{Hill}}*\log\left(\frac{100}{100-1*\mathrm{Min}}\right))\right) \end{array}$$

The medians of the quantitative variables were compared across different groups via the Mann–Whitney test and the ggpubr R package.

## Results

### IFNα2b and IFNβ1a decrease the proliferation of MIAPaCa‐2 and PANC‐1 cells

**Fig. 1:**
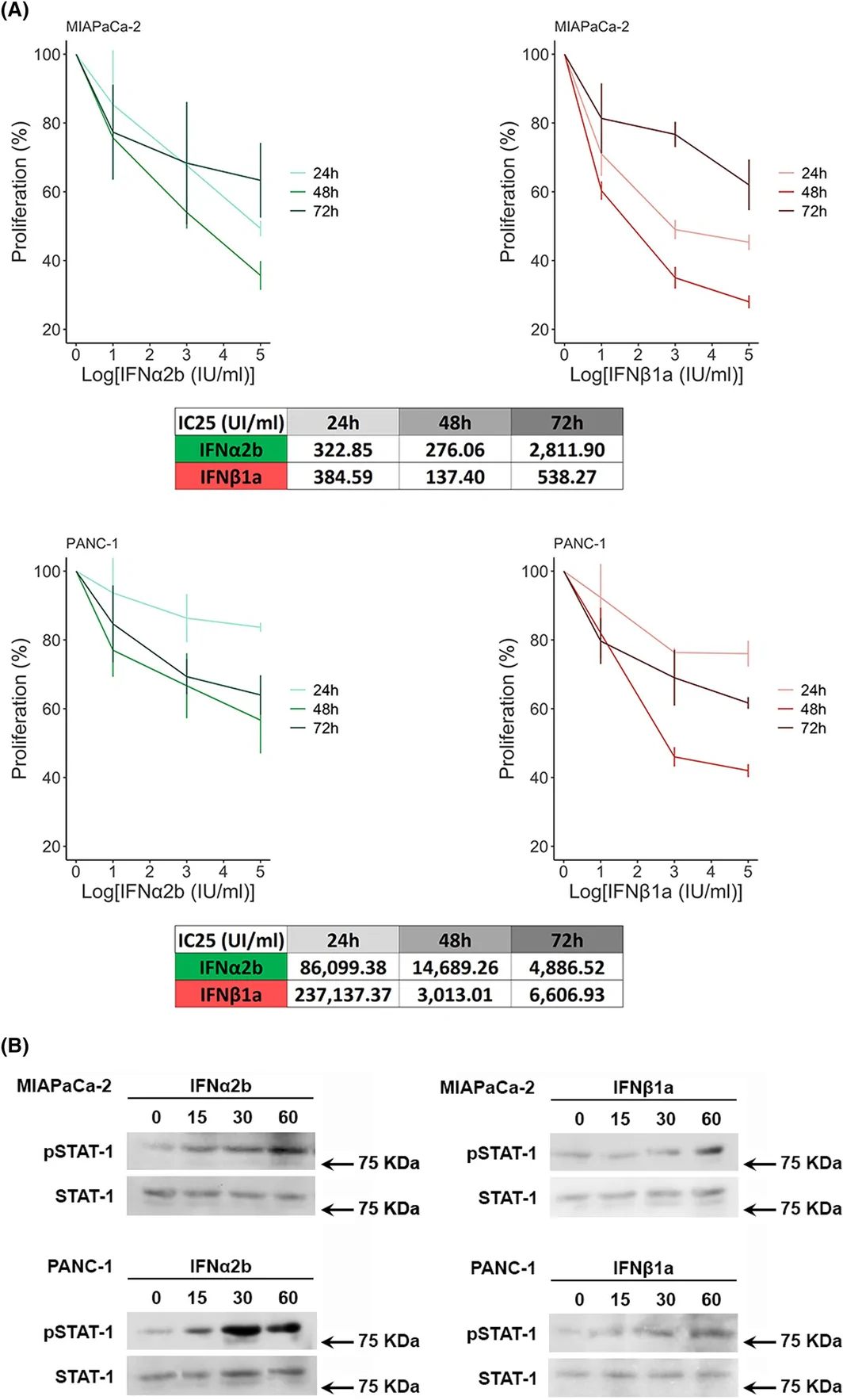
Effects of IFNα2b and IFNβ1a on cell proliferation and STAT1 phosphorylation. Cell proliferation of MIAPaCa‐2 and PANC‐1 cells (A) was determined by [^3^H]TdR incorporation after 24, 48, and 72 h of treatment. The results are expressed as the percentage of [^3^H]TdR incorporation in relation to the vehicle control, as described in the ‘Materials and methods’ section. The dose–response curves are shown for the MIAPaCa‐2 and PANC‐1 cell lines, along with their IC25 values. (B) Representative western blots showing phosphorylated STAT1 (pSTAT1) and total STAT1 in MIAPaCa‐2 and PANC‐1 cells treated with 1000 UI/ml of IFNα2b or IFNβ1a for 0, 15, 30, and 60 min. In the graphs, each dot represents the mean ± SD of at least 3 independent experiments.

IFN‐I is associated with beneficial effects against tumors, principally because of its ability to inhibit proliferation, induce apoptosis, and modulate the tumor microenvironment. In this context, the antitumor effects of IFNα2b have been widely studied over the past decade. Although the antitumor effects of IFNα2b have been extensively studied, the effects of IFNβ1a on pancreatic cancer cells are less well characterized. We first investigated the antiproliferative effects of both IFNs in MIAPaCa‐2 and PANC‐1 cells. The results showed that the inhibition of proliferation was time‐ and dose‐dependent manner, although the inhibition remained incomplete even at the highest concentrations tested. Therefore, the IC25 values were calculated becauseIC50 values could not be determined under all experimental conditions (Fig. 1, and Table S1). At 24 h, no differences were detected between the IC25 values of the two IFNs (Fig. 1A). However, the IC25 for IFNα2b was 2.01‐fold greater than the IC25 for IFNβ1a after 48 h of treatment in MIAPaCa‐2 cells, indicating that IFNβ1a exerted a stronger antiproliferative effect. In PANC‐1 cells, the IC25 for IFNα2b was 4.88‐fold higher than for IFNβ1a, again indicating a greater potency of IFNβ1a at this time point. Interestingly, the antiproliferative effect observed at 72 h were less effective than those observed at 48 h, suggesting the emergence of a less responsive cell population that continued to proliferate. To verify the engagement of the canonical type I interferon signaling pathway, we analyzed STAT1 phosphorylation and total STAT1 levels in MIAPaCa‐2 and PANC‐1 cells treated with IFNα2b or IFNβ1a. Both interferons induced STAT1 Tyr701 phosphorylation, confirming the activation of downstream STAT1 signaling in both pancreatic cancer cell lines (Fig. 1B). These findings support the interpretation that the antiproliferative effects observed in these cells occurred in response to biologically active type I interferon treatment.

### Differential effects of IFNα2b and IFNβ1a on the response of pancreatic cancer cells to gemcitabine

Considering that pancreatic tumors are highly resistant to gemcitabine and that type I IFNs have antiproliferative effects on pancreatic cancer cells, we investigated the response of MIAPaCa‐2 and PANC‐1 cells to combined treatment with IFNα2b or IFNβ1a and gemcitabine.

**Fig. 2:**
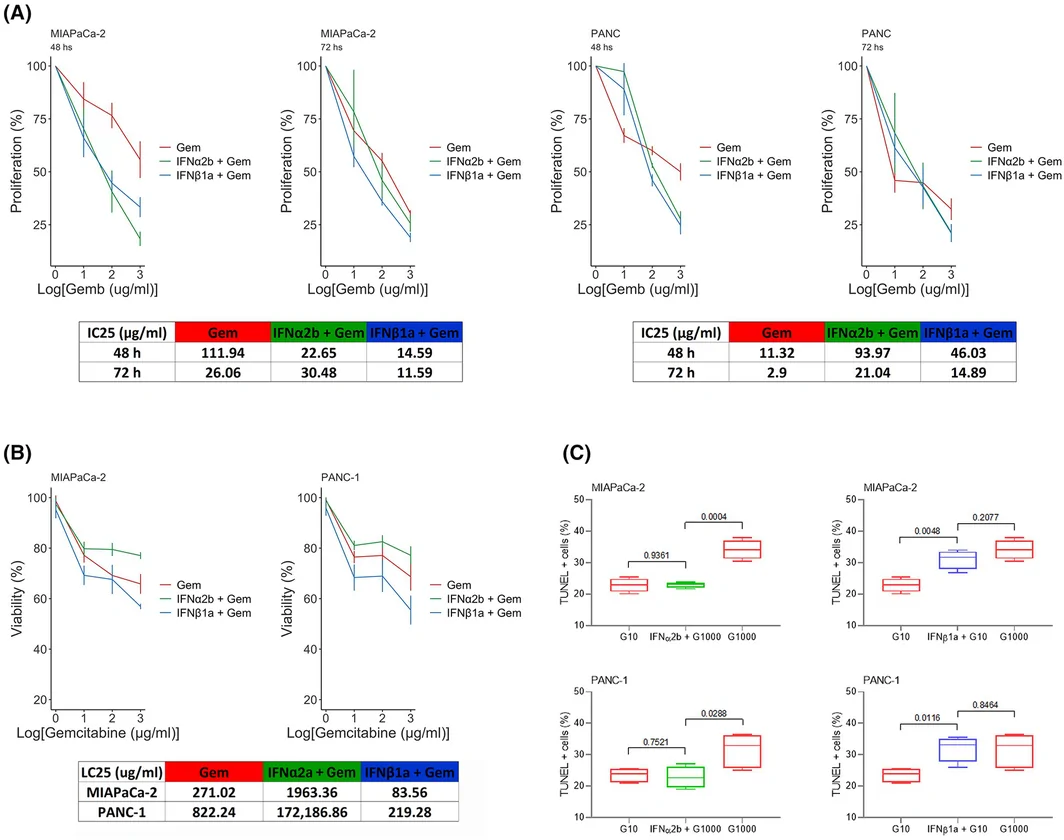
Effects of IFNα2b and IFNβ1a on the cellular response to gemcitabine. (A) Cell proliferation was determined by [^3^H]TdR incorporation after 48 and 72 h of treatment. The results are expressed as the percentage of [^3^H]TdR incorporation in relation to the vehicle control, as described in the ‘Materials and methods’ section. The dose–response curves are shown for the MIAPaCa‐2 and PANC‐1 cell lines, along with their IC25 values. (B) Cell death was evaluated via a TUNEL assay, and the percentage of viable cells was calculated as (% of total cells – TUNEL‐positive cells). The dose–response curves are shown for the MIAPaCa‐2 and PANC‐1 cell lines, along with their LC25 values. In all the graphs, each dot represents the mean ± SD of at least 3 independent experiments. (C) Comparison of gemcitabine‐induced cell death in the presence or absence of IFNs. The results are expressed as the percentage of TUNEL‐positive cells in relation to the vehicle control, as described in the ‘Materials and methods’ section. Box plots showing the different doses of gemcitabine with or without IFNα2b or IFNβ1a in MIAPaCa‐2 and PANC‐1 cells. Statistical significance was determined using an unpaired two‐tailed Student's *t*‐test (95% confidence level). Data are presented as box‐and‐whisker plots, where the central horizontal line indicates the median, the box limits represent the first (25th percentile) and third (75th percentile) quartiles, and the whiskers represent the minimum and maximum values.

First, we evaluated the cytostatic effect of each combination. The results obtained were somewhat different between the two cell lines. In the case of MIAPaCa‐2 (Fig. 2A) cells, the IC25 of IFNα2b was 4.94‐fold lower than the IC25 of gemcitabine at 48 h and 1.17‐fold greater at 72 h. Similarly, the IC25 of IFNβ1a was 7.67‐fold lower than the IC25 of gemcitabine at 48 h and 2.25‐fold lower at 72 h. These results suggest that, in general, the combination of both IFN‐I and gemcitabine is more effective in inhibiting MIAPaCa‐2 cell proliferation. In contrast, none of the combinations of IFN‐I with gemcitabine inhibited the proliferation of PANC‐1 cells (Fig. 2B) more efficiently than did gemcitabine alone (Table S2A).

Next, we investigated whether combining IFNα2b and IFNβ1a with gemcitabine could trigger different percentages of cell death than gemcitabine alone. Surprisingly, opposite responses were observed for both IFN‐Is. Cell viability was greater when the cells were treated with IFNα2b + gemcitabine than when they were treated with gemcitabine alone. Moreover, the combination of IFNβ1a + gemcitabine was more lethal. The LC25 of IFNα2b + gemcitabine was 7.24‐fold greater, and the LC25 of IFNβ1a + gemcitabine was 3.24‐fold lower than the LC25 of gemcitabine in MIAPaCa‐2 cells (Fig. 2C‐I). Moreover, in PANC‐1 (Fig. 2C‐II) cells, the differences were greater, with an LC25 of IFNα2b + gemcitabine of 209.40‐fold higher and an LC25 of IFNβ1a + gemcitabine of 3.75‐fold lower than the LC25 of gemcitabine.

The maximum values of dead cells obtained also differed depending on each treatment. Gemcitabine induced 34.17 ± 3.75% of the MIAPaCa‐2 TUNEL+ cells, whereas 22.95 ± 1.17% and 43.10 ± 0.87% of the MIAPaCa‐2 TUNEL+ cells were induced by the combination of IFNα2b and IFNβ1a, respectively. Similarly, gemcitabine induced 31.32 ± 5.33% of PANC‐1 TUNEL+ cells, and the combination of IFNα2b and IFNβ1a triggered 22.85 ± 3.40% and 44.52 ± 5.55%, respectively (Table S2B).

Moreover, the presence of IFNα2b reduced the lethal effect of 1000 μg/mL of gemcitabine to the values observed at a dose of 10 μg/mL in both cell lines, whereas the presence of IFNβ1a increased the lethal effect at the lower doses tested, 10 μg/mL, to similar values obtained at a dose of 1000 μg/mL, supporting the hypothesis that IFNβ1a sensitizes cells to chemotherapy, whereas IFNα2b increases their resistance (Fig. 2C).

### IFNα2b and IFNβ1a differentially modulate autophagosome biogenesis and autophagy flux

**Fig. 3:**
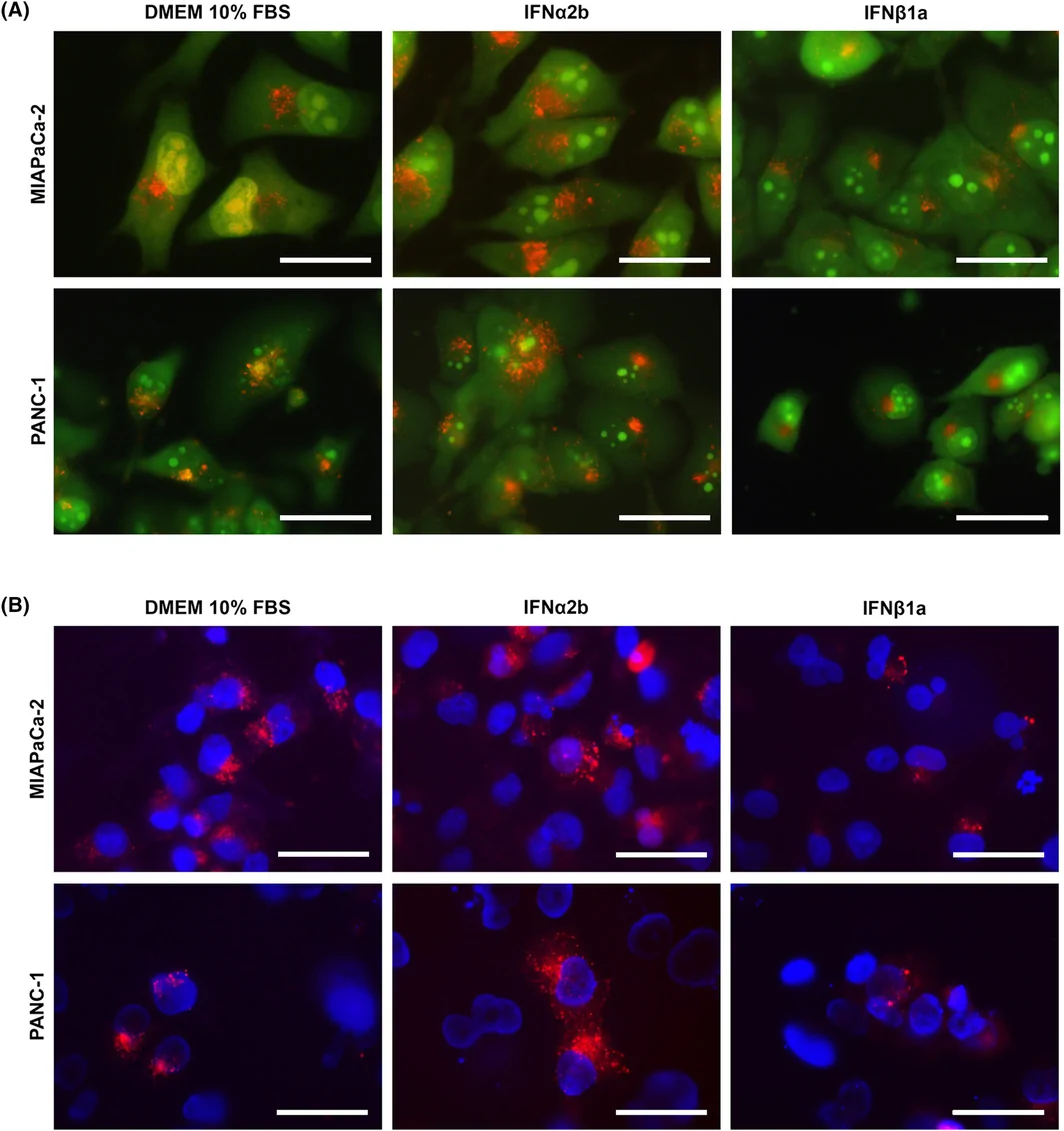
Qualitative assessment of acidic vesicular organelles and LC3‐positive structures following type I IFN treatment. (A) Representative fluorescence microscopy images of acridine orange‐stained MIAPaCa‐2 and PANC‐1 cells cultured in DMEM containing 10% FBS or treated with 1000 IU/mL IFNα2b or IFNβ1a for 24 h. (B) Representative fluorescence microscopy images of MIAPaCa‐2 and PANC‐1 cells transfected with an RFP‐LC3 plasmid and cultured under the same treatment conditions. Qualitative inspection showed an increased number of acidic vesicular organelles and RFP‐LC3‐positive puncta following IFNα2b treatment, whereas IFNβ1a treatment was associated with a lower number of these structures. Images were acquired at 400× magnification. Scale bars, 75 μm.

To explore the reason behind the opposing effects observed between the two type I IFNs, we questioned whether autophagy could be involved. To that end, we conducted a series of assays to determine whether type I IFNs were able to modulate autophagy flux. First, acidic vesicular organelles were visualized by acridine orange staining (Fig. 3A), and LC3‐positive vesicular structures were examined after transfection with an RFP‐LC3 plasmid (Fig. 3B). Qualitative microscopic analysis showed that IFNα2b increased the number of acidic vesicles and RFP‐LC3‐positive puncta, whereas IFNβ1a reduced these structures in both cell lines.

**Fig. 4:**
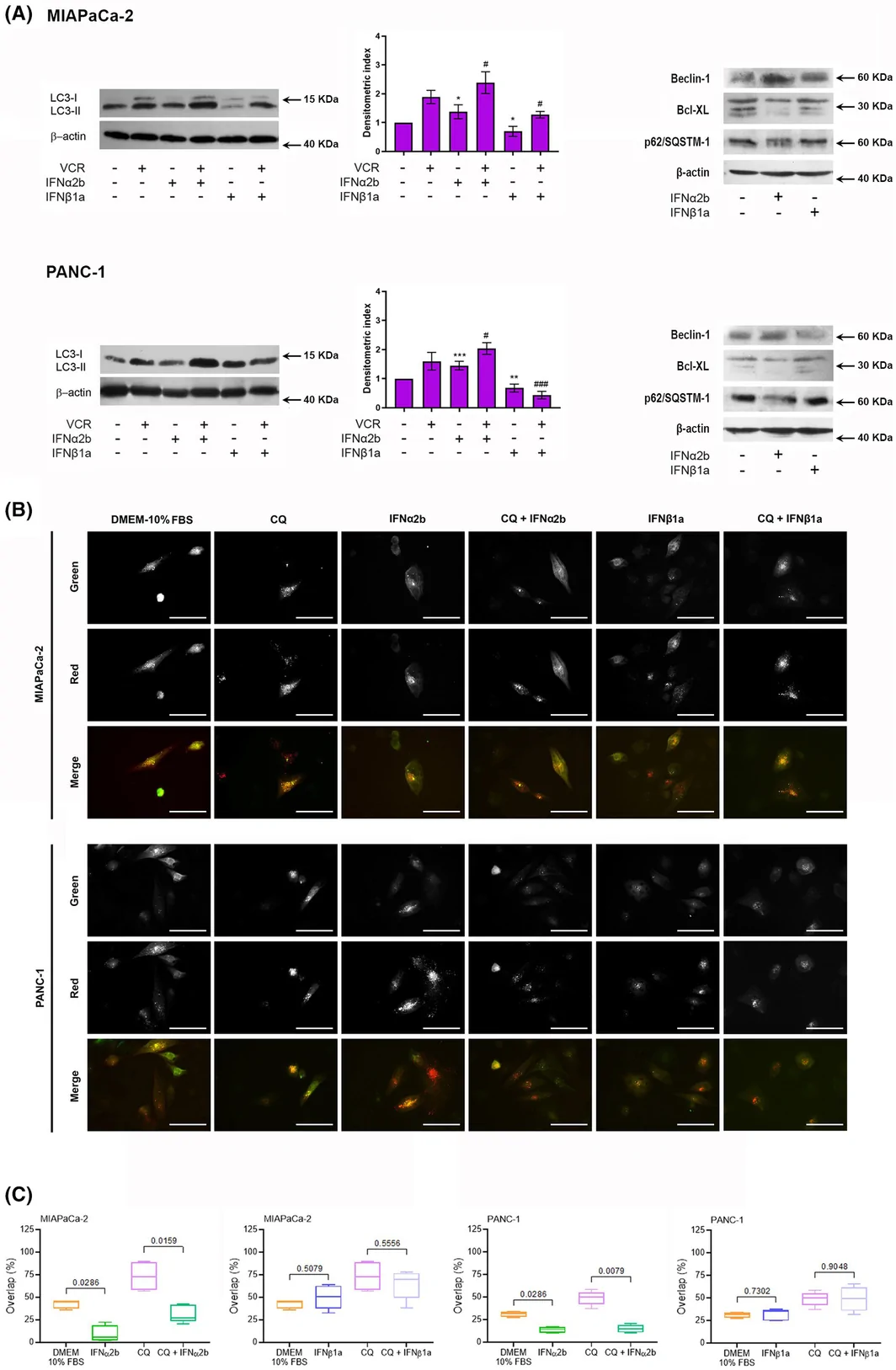
Modulation of autophagy flux by type I IFNs. Endogenous LC3‐II levels were evaluated by western blotting in MIAPaCa‐2 and PANC‐1 cells under basal conditions and after treatment with 1000 UI/ml IFNα2b/IFNβ1a for 24 h and/or 10 μm VCR for the last 6 h. Beclin‐1, Bcl‐XL, p62/SQSTM1, and β‐actin levels were also analyzed after 24 h of treatment with IFNα2b or IFNβ1a. The bars show the densitometric indices ± SDs of 3 independent experiments. Statistical comparison vs basal condition is indicated with asterisks (*), and vs VCR with hash symbols (#) */#*P* < 0.05; **P < 0.01; ####*P* < 0.0001. (B) Representative images of MIAPaCa‐2 and PANC‐1 cells transfected with the pBABE‐puro‐mCherry‐EGFP‐LC3B plasmid. The cells were cultured in the presence of 1000 UI/ml IFNα2b or IFNβ1a for 24 h and/or 25 μm chloroquine (CQ) for the last 6 h. Images were obtained at 400x magnification. Scale bars correspond to 75 μm. (C) Quantification of the mCherry/EGFP overlap area in MIAPaCa‐2 and PANC‐1 cells under the indicated treatment conditions. The overlap area is expressed as a percentage, and the corresponding *P* values are shown. Statistical significance was determined using an unpaired two‐tailed Student's *t*‐test (95% confidence level). Data are presented as box‐and‐whisker plots, where the central horizontal line indicates the median, the box limits represent the first (25th percentile) and third (75th percentile) quartiles, and the whiskers represent the minimum and maximum values.

Next, LC3‐II levels were determined in the presence of IFNs and/or vincristine (VCR), a microtubule network inhibitor that blocks transport to lysosomes. VCR is expected to inhibit the degradation of autophagosomes [34]. For this purpose, the cells were exposed to 1000 IU/mL IFNα2b or IFNβ1a for 24 h and/or 10 μm VCR for the last 6 h. Compared with untreated cells, VCR inhibited LC3 degradation, resulting in the accumulation of LC3‐II in cells treated with IFNα2b (Fig. 4A). Importantly, in IFNα2b‐treated cells, the changes in LC3‐II levels were greater in the presence of VCR than those in cells treated with VCR alone, suggesting that IFNα2b stimulates autophagic flux. In contrast, the levels of LC3‐II in cells treated with IFNβ1a + VCR were lower than those in cells treated with VCR alone, indicating that IFNβ1a inhibited the formation of autophagosomes.

To further characterize these responses, the protein levels of Beclin‐1, Bcl‐XL, and p62/SQSTM1 were analyzed by western blotting after 24 h of treatment with 1000 IU/mL IFNα2b or IFNβ1a in MIAPaCa‐2 and PANC‐1 cells (Fig. 4A). IFNα2b increased Beclin‐1 protein levels and decreased the levels of Bcl‐XL and p62/SQSTM1 in both cell lines. In contrast, IFNβ1a produced the opposite pattern, with lower Beclin‐1 levels and higher levels of Bcl‐XL and p62/SQSTM1. These changes are consistent with enhanced autophagosome biogenesis and autophagic flux following IFNα2b treatment, whereas the response to IFNβ1a is consistent with reduced autophagosome biogenesis and impaired autophagic processing. Together with the LC3‐II and fluorescence microscopy analyses, these findings support the differential regulation of autophagy by the two types I IFNs. To confirm and further assess these results, we transfected cells with the plasmid pBABE‐puro‐mCherry‐EGFP‐LC3B (Addgene #22418), which expresses a chimeric LC3 with GFP (green) and mCherry (red). Since GFP is sensitive to autolysosomal acidity and loses fluorescence, while mCherry remains stable, vesicles emitting both colors appear yellow and correspond to autophagosomes. Those emitting only red correspond to autolysosomes (Fig. 4B). Cells were cultured in the presence of IFNα2b or IFNβ 1000 IU/mL for 24 h and/or 25 μm chloroquine (CQ), a proton pump inhibitor, for the last 6 h. Photographs were subsequently taken, and the percentage of overlap between red and green fluorescence was analyzed via fiji software. A greater percentage of cells exhibiting color overlap indicated a greater proportion of vesicles containing both GFP and mCherry signals, consistent with reduced autophagosome maturation and/or lysosomal degradation under these experimental conditions. In contrast, cells with predominantly red vesicles presented increased autophagic flux and/or enhanced degradation.

As shown in Fig. 4C, IFNα2b decreased the overlap area between both colors, resulting in cells with a higher content of red vesicles (autolysosomes) in both cell lines.

Additionally, combining IFNα2b with CQ yielded lower overlap values than those observed with CQ treatment alone. The decrease in the number of yellow vesicles (early autophagosomes) suggests that IFNα2b enhances autophagic flux, a finding that is consistent with the analysis of LC3‐II by western blot (Fig. 4A). Conversely, IFNβ1a did not result in changes in the overlap area compared with the basal condition or in combination with CQ compared with CQ treatment alone (Fig. 4C). Moreover, we evaluated the effect of IFNα2b and IFNβ1a on gemcitabine‐induced autophagy (Fig. S1). We observed that IFNα2b enhanced gemcitabine‐induced autophagy, whereas IFNβ1a counteracted this process. These results, in accordance with the analysis of LC3 levels by western blot, suggest that IFNβ1a does not alter autophagic flux but inhibits autophagosome formation.

### 3‐MA reverses IFNα2b‐mediated resistance to gemcitabine‐induced cell death, whereas IFNα2b prevents IFNβ1a‐mediated sensitization

**Fig. 5:**
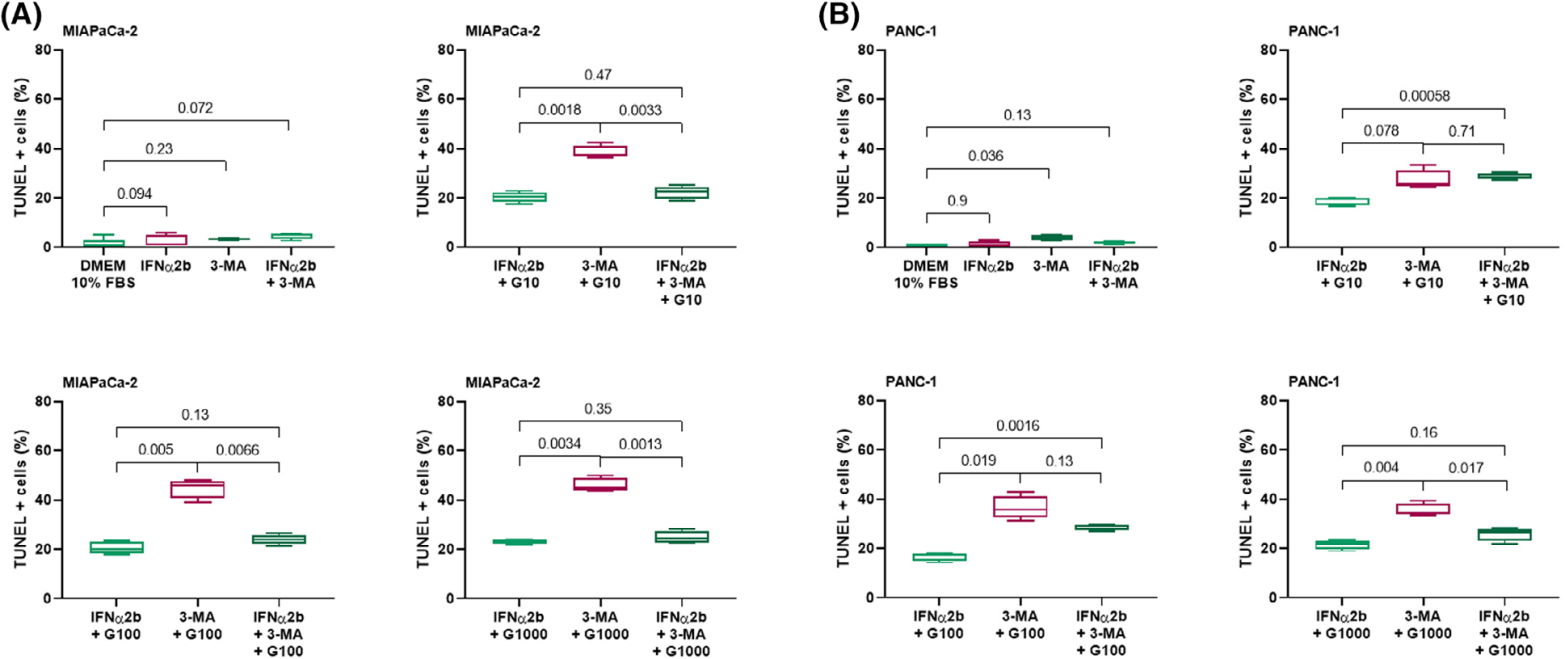
Rescue of resistance to IFNα2b + gemcitabine‐induced cell death by 3‐MA. Cell death was evaluated via the TUNEL assay, and the results were calculated as described in the ‘Materials and Methods’. Box plots showing percentages of cell death obtained for MIAPaCa‐2 (A) and PANC‐1 (B) cells. Statistical significance was determined using an unpaired two‐tailed Student's *t*‐test (95% confidence level). Data are presented as box‐and‐whisker plots, where the central horizontal line indicates the median, the box limits represent the first (25th percentile) and third (75th percentile) quartiles, and the whiskers represent the minimum and maximum values.

Previously, we reported that autophagy is one of the mechanisms responsible for chemotherapy failure and that inhibiting this process with 3‐MA sensitized MIAPaCa‐2 and PANC‐1 cells to gemcitabine [4]. To determine whether autophagy contributed to the differences in cell death observed after combining gemcitabine with IFNα2b or IFNβ1a, we performed rescue assays. First, we evaluated whether the resistance to cell death observed after treatment with IFNα2b plus gemcitabine could be reversed by preincubation with 3‐MA, an autophagy inhibitor. For this purpose, 10 mm 3‐MA was added to the cultures 1 h before the addition of IFNα2b plus gemcitabine. For all doses of gemcitabine tested, the IFNα2b‐mediated resistance was completely reversed in MIAPaCa‐2 cells (Fig. 5A). The resistance induced by IFNα2b was not reversed when IFNα2b was combined with 10 μg/mL gemcitabine; the reversal was partial when IFNα2b was combined with 100 μg/mL gemcitabine and complete when it was combined with 1000 μg/mL gemcitabine.

**Fig. 6:**
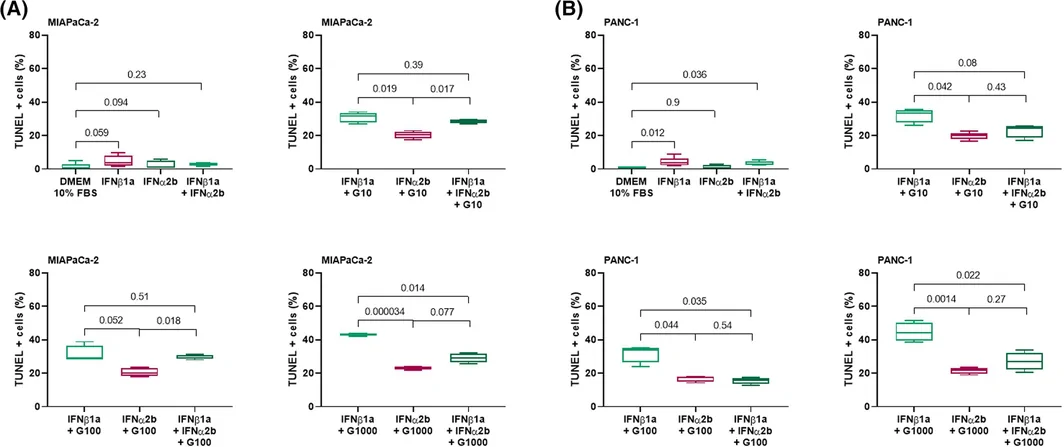
IFNα2b prevents sensitization to gemcitabine induced by IFNβ1a. Cell death was evaluated via the TUNEL assay, and the results were calculated as described in the ‘Materials and Methods’. Box plots showing percentages of cell death obtained for MIAPaCa‐2 (A) and PANC‐1 (B) cells. Statistical significance was determined using an unpaired two‐tailed Student's *t*‐test (95% confidence level). Data are presented as box‐and‐whisker plots, where the central horizontal line indicates the median, the box limits represent the first (25th percentile) and third (75th percentile) quartiles, and the whiskers represent the minimum and maximum values.

Next, we evaluated whether IFNα2b could prevent the sensitizing effect of IFNβ1a on gemcitabine‐induced cell death. For this purpose, IFNα2b was added to the cell cultures 1 h before the addition of IFNβ1a plus gemcitabine. In MIAPAcA‐2 cells, the sensitizing effect of IFNβ1a was not prevented when combined with 10 μg/mL or 100 μg/mL gemcitabine, but it was completely prevented when 1000 μg/mL gemcitabine was used (Fig. 6A). This effect was more pronounced in PANC‐1 cells, in which IFNα2b completely prevented IFNβ1a‐induced sensitization to gemcitabine‐induced cell death (Fig. 6B).

## Discussion

The present study reveals distinct functional divergence between the two types I interferons examined here, IFNα2b and IFNβ1a, in their antitumor activity against pancreatic cancer cells, with important implications for the response to gemcitabine. Our data showed that while both interferons exhibit time‐ and dose‐dependent cytostatic effects, IFNβ1a displays superior potency compared to IFNα2b, particularly at 48 h of treatment, where its IC25 values were 2.01‐fold and 4.88‐fold lower in MIAPaCa‐2 and PANC‐1 cells, respectively. These findings are consistent with previous reports identifying IFN‐β as a more potent antiproliferative agent than IFN‐α in pancreatic cancer models [35]. STAT1 phosphorylation was detected following treatment with both IFNs, confirming the engagement of downstream type I IFN signaling. However, the present data do not establish that the magnitude of STAT1 phosphorylation alone accounts for the greater antiproliferative potency of IFNβ1a.

Despite these differences, neither IFNα2b nor IFNβ1a achieved complete growth inhibition, even at high doses, suggesting the presence of a less responsive cell population. This observation aligns with the well‐documented refractory nature of pancreatic adenocarcinoma to interferon‐based monotherapy. Notably, the divergent biological effects of IFNα2b and IFNβ1a occur despite both cytokines signaling through the same IFNAR1/2 receptor complex, a phenomenon previously described in other tumor models. This phenomenon can be explained, at least in part, by their distinct receptor‐binding affinities and downstream signaling kinetics. IFNβ binds IFNAR with an approximately 50‐fold higher affinity than IFNα, which results in a stronger and more sustained activation of JAK–STAT signaling and a distinct pattern of interferon‐stimulated gene (ISG) expression [32]. These differences in receptor engagement and signaling duration can lead to divergent cellular responses, including opposite effects on proliferation, apoptosis, and autophagy.

Clinically and experimentally, IFNα has been the most extensively studied interferon. When combined with other agents, such as 5‐fluorouracil, cisplatin, retinoic acid, or leucovorin, IFNα has been reported to enhance therapeutic efficacy in pancreatic cancer [30, 36, 37, 38, 39, 40]. In line with this, Iwahashi *et al*. demonstrated *the in vitro* antiproliferative effect of a combination of valproic acid with gemcitabine and pegylated IFNα2b on pancreatic cell lines; however, the capacity of this combined therapy to induce cell death has not been tested [41]. In contrast, data on the effects of IFNβ in pancreatic cancer remain limited. Nevertheless, a growth‐inhibitory effect of IFNβ in combination with gemcitabine has been reported *in vitro* [32], supporting a potential therapeutic role for this interferon subtype.

The most striking finding of our investigation concerns the opposing effects of these interferons on gemcitabine‐induced cytotoxicity. While IFNβ1a enhanced gemcitabine‐mediated cell death, IFNα2b conferred marked resistance, increasing the LC25. Although these clinical observations are not directly comparable to the present *in vitro* findings, this paradoxical behavior mirrors clinical observations where IFN‐α‐based adjuvant chemoradiation improved 5‐year survival to 55% in selected patients, yet broader applications have yielded disappointing results due to tumor resistance [40].

Central to our observation is the differential modulation of autophagy. We demonstrate that IFNα2b stimulates autophagic flux, as evidenced by greater LC3‐II accumulation in the presence of vincristine, consistent with enhanced autophagic flux, and reduced red/green fluorescence overlap in mCherry‐EGFP‐LC3B reporter assays. This enhanced autophagy contributes to chemoresistance, as supported by the partial or complete reversal of IFNα2b‐induced resistance with 3‐methyladenine (3‐MA), dependent on the cell line and gemcitabine dose. These findings are consistent with established literature demonstrating that autophagy serves as a critical survival mechanism in pancreatic cancer cells, protecting them from gemcitabine‐induced apoptosis [4, 42]. The protective role of autophagy in interferon‐treated cells has been previously reported in breast cancer models, where IFN‐β‐induced autophagy counteracted its proapoptotic function [43]. In our model, IFNβ1a reduced LC3‐II accumulation in the presence of vincristine, consistent with inhibition of autophagosome formation, but did not produce clear changes in autophagic flux in the tandem mCherry‐EGFP‐LC3B reporter assay. This reduction in autophagosome formation was associated with sensitization to gemcitabine, an effect that was prevented by IFNα2b in a cell line‐ and gemcitabine dose‐dependent manner, further supporting the antagonistic interplay between these interferon subtypes. The rescue of IFNα2b‐mediated resistance by 3‐MA supports a functional contribution of autophagy to this phenotype, providing a rationale for combination therapies targeting both interferon signaling and autophagy. This approach could potentially address one limitation of type I interferon therapy—tumor resistance—by exploiting the differential regulation of survival pathways by IFNα2b and IFNβ1a.

Despite the enhanced antiproliferative and cytotoxic effects observed with IFNβ–gemcitabine combinations compared with gemcitabine alone, the overall response observed *in vitro* remains incomplete, as none of the treatment conditions resulted in complete elimination of the tumor cell population. Even at higher drug concentrations and prolonged exposure times, a significant fraction of cells remained viable, consistent with the involvement of intrinsic or adaptive resistance mechanisms that limit the efficacy of chemotherapy, even when combined with type I interferon signaling. These findings highlight that increased cytotoxic activity does not necessarily translate into full tumor cell killing *in vitro*.

In this context, a deeper understanding of the cellular mechanisms engaged by pancreatic cancer cells to withstand chemotherapeutic stress—such as activation of survival pathways and protective stress responses—is essential. Elucidating these resistance mechanisms is critical not only to explain the limited efficacy of current treatment strategies but also to provide a rational basis for the development of improved therapeutic combinations aimed at overcoming chemoresistance in pancreatic ductal adenocarcinoma.

Future studies should focus on dissecting the upstream signaling events that differentiate IFNα2b from IFNβ1a, including potential differences in Tyk2 and Jak1 activation, STAT dimerization, and nuclear translocation. Ultimately, a more comprehensive understanding of these mechanisms will be essential for optimizing interferon‐based therapeutic strategies and improving outcomes in pancreatic cancer, a disease that remains notoriously refractory to conventional treatments.

Together, these findings underscore that a precise understanding of subtype‐specific type I interferon signaling and its impact on autophagy‐driven survival pathways is critical for the rational design of combination therapies aimed at overcoming chemoresistance in pancreatic ductal adenocarcinoma.

## Conclusion

This study demonstrates that type I interferons exert opposing effects on autophagy and chemosensitivity in pancreatic ductal adenocarcinoma cells. Specifically, IFNα2b induces autophagy and confers protection against gemcitabine‐induced apoptosis, whereas IFNβ1a inhibits autophagosome formation and enhances gemcitabine‐mediated cytotoxicity. These findings indicate that subtle differences in type I interferon signaling critically influence cell fate and therapeutic outcome. The observation that autophagy inhibition restores gemcitabine sensitivity in IFNα2b‐treated cells, while autophagy induction counteracts IFNβ1a‐driven sensitization, underscores the central role of autophagy in modulating drug response. Overall, our results identify IFNβ1a as a potential chemosensitizing agent in pancreatic cancer and provide a rationale for combining autophagy‐targeting strategies with standard chemotherapy to overcome resistance and improve treatment efficacy.

## Conflicts of interest

The authors declare no conflicts of interest.

## Author contributions

LEB, SAB, MAP, MLP, and DLP performed most of the experiments. SLL performed the cell death experiments and edited the manuscript. MML, MAP, JG, DHG, MNG, and EA contributed to the design of the study and edited and supervised the manuscript. MML, SLL, and DLP analyzed the data and wrote the manuscript. DLP contributed to funding acquisition. All the authors contributed to the work and have read and approved the final manuscript.

## Supporting information


**Table S1.** Effect of IFNα2b and IFNβ1a on cell proliferation of MIAPaCa‐2 (A) and PANC‐1 (B). Cell proliferation was determined by [3H]TdR incorporation after 24, 48 and 72 h of treatment. Results are expressed as the percentage of cell proliferation, as described in the ‘Materials and methods’ section. The table shows the percentages of cell proliferation ± SD of at least 3 independent experiments.
**Table S2.** Effect of IFNα2b and IFNβ1a on cellular response to gemcitabine. (A) Cell proliferation was determined by [3H]TdR incorporation after 48 and 72 h of treatment. The table shows the percentages of cell proliferation ± SD of at least 3 independent experiments. (B) Cell death was evaluated after 72 h by TUNEL assay and results were calculated as described in ‘materials and Methods’. The table shows the percentages of TUNEL+ cell ± SD of at least 3 independent experiments.
**Fig. S1**. Modulation of autophagy flux by type I IFNs. (A) Representative images of MIAPaCa‐2 and PANC‐1 cells transfected with the pBABE‐puro‐mCherry‐EGFP‐LC3B plasmid. The cells were cultured in the presence of 1000 UI/ml IFNα2b /IFNβ1a and/or 1000 μg/mL Gemcitabine. Images were obtained at 400x magnification. White bars correspond to 75 μm. (D) Graphs of the MIAPaCa‐2 and PANC‐1 cell lines showing the RFP/GFP overlap area of the different treatments expressed as a percentage, p values are indicated. Statistical significance was determined using an unpaired two‐tailed Student's *t*‐test (95% confidence level). Data are presented as box‐and‐whisker plots, where the central horizontal line indicates the median, the box limits represent the first (25th percentile) and third (75th percentile) quartiles, and the whiskers represent the minimum and maximum values.

## Data Availability

The datasets used and/or analyzed during the current study are available from the corresponding author on reasonable request.
