# Supplementary material for: Type I interferons modulate autophagy to shape gemcitabine response in pancreatic cancer cells
Source: FEBS Open Bio. 2026 Sep 15:10.1002/2211-5463.70342. Online ahead of print. doi: 10.1002/2211-5463.70342 (PMC13576371; doi:10.1002/2211-5463.70342)
Supplement: Supplementary file 1 [file FEB4-9999-0-s001.docx]

**Supporting information**

**Supplementary table 1:** Effect of IFNα2b and IFNβ1a on cell proliferation of MIAPaCa-2 (A) and PANC-1 (B). Cell proliferation was determined by [3H]TdR incorporation after 24, 48 and 72h of treatment. Results are expressed as the percentage of cell proliferation, as described in the “Materials and methods” section. The table shows the percentages of cell proliferation ± SD of at least 3 independent experiments.

**(A) MIAPaCa-2**

| **LOG[IFNα2b (UI/ml)]** | **24h** | **48h** | **72h** |
| --- | --- | --- | --- |
| 0 | 100.00 ± 0.00 | 100.00 ± 0.00 | 100.00 ± 0.00 |
| 1 | 85.33 ± 15.63 | 75.67 ± 2.08 | 77.33 ± 13.65 |
| 3 | 67.67 ± 8.14 | 54.00 ± 4.59 | 68.33 ± 17.62 |
| 5 | 49.33 ± 2.08 | 35.67 ± 4.04 | 63.33 ± 10.69 |

| **LOG[IFNβ1a (UI/ml)]** | **24h** | **48h** | **72h** |
| --- | --- | --- | --- |
| 0 | 100.00 ± 0.00 | 100.00 ± 0.00 | 100.00 ± 0.00 |
| 1 | 71.00 ± 6.24 | 60.33 ± 2.52 | 81.33 ± 10.02 |
| 3 | 49.00 ± 2.65 | 35.00 ± 3.00 | 76.67 ± 3.51 |
| 5 | 45.33 ± 2.08 | 28.00 ± 1.73 | 62.0 ± 7.21 |

**(B) PANC-1**

| **LOG[IFNα2b (UI/ml)]** | **24h** | **48h** | **72h** |
| --- | --- | --- | --- |
| 0 | 100.00 ± 0.00 | 100.00 ± 0.00 | 100.00 ± 0.00 |
| 1 | 93.67 ± 10.02 | 77.00 ± 7.55 | 84.67 ± 11.02 |
| 3 | 86.33 ± 6.81 | 66.67 ± 9.29 | 69.33 ± 4.93 |
| 5 | 83.67 ± 1,15 | 56.67 ± 9.50 | 64.00 ± 5.57 |

| **LOG[IFNβ1a (UI/ml)]** | **24h** | **48h** | **72h** |
| --- | --- | --- | --- |
| 0 | 100.00 ± 0.00 | 100.00 ± 0.00 | 100.00 ± 0.00 |
| 1 | 92.33 ± 9.61 | 82.00 ± 7.21 | 79.67 ± 6.51 |
| 3 | 76.33 ± 1.15 | 46.00 ± 2.65 | 69.00 ± 7.94 |
| 5 | 76.00 ± 3.61 | 42.00 ± 1.73 | 61.67 ± 1.53 |

**Supplementary table 2:** Effect of IFNα2b and IFNβ1a on cellular response to gemcitabine. (A) Cell proliferation was determined by [3H]TdR incorporation after 48 and 72h of treatment. The table shows the percentages of cell proliferation ± SD of at least 3 independent experiments. (B) Cell death was evaluated after 72h by TUNEL assay and results were calculated as described in “materials and Methods”. The table shows the percentages of TUNEL+ cell ± SD of at least 3 independent experiments.

**(A)**

**MIAPaCa-2**

| **LOG[Gem (µg/ml)]** |  | **48h** |  |  | **72h** |  |
| --- | --- | --- | --- | --- | --- | --- |
|  | **Gem** | **IFNα2b + Gem** | **IFNβ1a + Gem** | **Gem** | **IFNα2b + Gem** | **IFNβ1a + Gem** |
| 0 | 100.00 ± 0.00 | 100.00 ± 0.00 | 100.00 ± 0.00 | 100.00 ± 0.00 | 100.00 ± 0.00 | 100.00 ± 0.00 |
| 1 | 84.40 ± 7.74 | 70.33 ± 9.61 | 66.00 ± 8.89 | 69.43 ± 1.07 | 78.33 ± 19.66 | 57.33 ± 4.93 |
| 2 | 76.57 ± 5.82 | 40.67 ± 9.71 | 44.67 ± 3.79 | 55.13 ± 3.59 | 46.00 ± 8.19 | 36.00 ± 1.73 |
| 3 | 55.73 ± 8.48 | 18.33 ± 3.21 | 33.33 ± 4.51 | 30.50 ± 1.15 | 25.67 ± 3.79 | 19.00 ± 2.00 |

**PANC-1**

| **LOG[Gem (µg/ml)]** |  | **48h** |  |  | **72h** |  |
| --- | --- | --- | --- | --- | --- | --- |
|  | **Gem** | **IFNα2b + Gem** | **IFNβ1a + Gem** | **Gem** | **IFNα2b + Gem** | **IFNβ1a + Gem** |
| 0 | 100.00 ± 0.00 | 100.00 ± 0.00 | 100.00 ± 0.00 | 100.00 ± 0.00 | 100.00 ± 0.00 | 100.00 ± 0.00 |
| 1 | 67.13 ± 3.25 | 97.33 ± 1.53 | 89.00 ± 12.12 | 45.93 ± 5.55 | 68.33 ± 18.72 | 61.33 ± 7.02 |
| 2 | 60.00 ± 1.90 | 53.00 ± 1.00 | 46.00 ± 2.65 | 44.87 ± 1.63 | 43.33 ±10.79 | 42.67 ± 8.62 |
| 3 | 49.97 ± 3.86 | 27.67 ± 3.51 | 24.67 ± 4.04 | 32.37 ± 4.86 | 21.33 ± 3.79 | 21.00 ± 4.00 |

**(B)**

**MIAPaCa-2**

| **LOG[Gem (μg/ml)]** | **Gem** | **Gem + IFNα2b** | **Gem + IFNβ1a** |
| --- | --- | --- | --- |
| 0 | 1.32 ± 2.03 | 1.32 ± 2.03 | 1.32 ± 2.03 |
| 1 | 22.83 ± 2.70 | 20.23 ± 2.61 | 30.77 ± 3.66 |
| 2 | 30.8 ± 1.04 | 20.52 ± 2.42 | 32.40 ± 5.60 |
| 3 | 34.17 ± 3.75 | 22.95 ± 1.17 | 43.10 ± 0.87 |

**PANC-1**

| **LOG[Gem (μg/ml)]** | **Gem** | **Gem + IFNα2b** | **Gem + IFNβ1a** |
| --- | --- | --- | --- |
| 0 | 0.84 ± 0.59 | 0.84 ± 0.59 | 0.84 ± 0.59 |
| 1 | 23.52 ± 2.25 | 18.93± 1.68 | 31.63 ± 5.01 |
| 2 | 22.90 ± 3.78 | 17.45 ± 2.40 | 31.07 ± 6.16 |
| 3 | 31.32 ± 5.33 | 22.85 ± 3.40 | 44.52 ± 5.55 |

**Supplementary Figure 1.** Modulation of autophagy flux by type I IFNs. (A) Representative images of MIAPaCa-2 and PANC-1 cells transfected with the pBABE-puro-mCherry-EGFP-LC3B plasmid. The cells were cultured in the presence of 1000 UI/ml IFNα2b /IFNβ1a and/or 1000 μg/ml Gemcitabine. Images were obtained at 400x magnification. White bars correspond to 75 μm. (D) Graphs of the MIAPaCa-2 and PANC-1 cell lines showing the RFP/GFP overlap area of the different treatments expressed as a percentage, p values are indicated. Statistical significance was determined using an unpaired two-tailed Student’s t-test (95% confidence level). Data are presented as box-and-whisker plots, where the central horizontal line indicates the median, the box limits represent the first (25th percentile) and third (75th percentile) quartiles, and the whiskers represent the minimum and maximum values.
